# Comprehensive Analysis of TIFY Transcription Factors and Their Expression Profiles under Jasmonic Acid and Abiotic Stresses in Watermelon

**DOI:** 10.1155/2019/6813086

**Published:** 2019-10-01

**Authors:** Youxin Yang, Golam Jalal Ahammed, Chunpeng Wan, Haoju Liu, Rongrong Chen, Yong Zhou

**Affiliations:** ^1^Jiangxi Key Laboratory for Postharvest Technology and Nondestructive Testing of Fruits & Vegetables, Collaborative Innovation Center of Postharvest Key Technology and Quality Safety of Fruits and Vegetables, College of Agronomy, Jiangxi Agricultural University, Nanchang 330045, China; ^2^College of Forestry, Henan University of Science and Technology, Luoyang 471023, China; ^3^College of Bioscience and Bioengineering, Jiangxi Agricultural University, Nanchang 330045, China; ^4^Key Laboratory of Crop Physiology, Ecology, and Genetic Breeding, Ministry of Education, Jiangxi Agricultural University, Nanchang 330045, China

## Abstract

The *TIFY* gene family is plant-specific and encodes proteins involved in the regulation of multiple biological processes. Here, we identified 15 *TIFY* genes in the watermelon genome, which were divided into four subfamilies (eight JAZs, four ZMLs, two TIFYs, and one PPD) in the phylogenetic tree. The *ClTIFY* genes were unevenly located on eight chromosomes, and three segmental duplication events and one tandem duplication event were identified, suggesting that gene duplication plays a vital role in the expansion of the *TIFY* gene family in watermelon. Further analysis of the protein architectures, conserved domains, and gene structures provided additional clues for understanding the putative functions of the TIFY family members. Analysis of qRT-PCR and RNA-seq data revealed that the detected *ClTIFY* genes had preferential expression in specific tissues. qRT-PCR analysis revealed that nine selected *TIFY* genes were responsive to jasmonic acid (JA) and abiotic stresses including salt and drought. JA activated eight genes and suppressed one gene, among which *ClJAZ1* and *ClJAZ7* were the most significantly induced. Salt and drought stress activated nearly all the detected genes to different degrees. These results lay a foundation for further functional characterization of *TIFY* family genes in *Citrullus lanatus*.

## 1. Introduction

TIFY transcription factors are plant-specific transcriptional regulators characterized by the presence of a highly conserved motif (TIF(F/Y)XG) in the TIFY domain (previously called ZIM domain) with a length of approximately 36 amino acids (aa) [1, 2]. According to their domain architectures, the TIFY proteins can be divided into four subfamilies, including TIFY, ZIM-like (ZML), jasmonate ZIM-domain (JAZ), and PEAPOD (PPD). Except for the TIFY subfamily, which only harbors the TIFY domain, members of other three subfamilies possess some additional domains [3]. For example, the ZML subfamily proteins possess a GATA zinc-finger DNA-binding domain and a CCT domain (CONSTANS, CO-like, and TOC1) [4, 5]. The JAZ subfamily proteins have a conserved jasmonic acid- (JA-) associated domain (Jas, also known as CCT-2 motif) with a SLX_2_FX_2_KRX_2_RX_5_PY (X represents any amino acid) consensus sequence, while the PPD subfamily proteins contain a typical PPD domain in the N-terminus and a truncated Jas motif lacking the conserved P and Y residues [6–8].

To date, a number of *TIFY* genes have been functionally characterized in *Arabidopsis* [9–12], rice [13–15], wheat [16, 17], tomato [18], cotton [19], and other plants [20–22]. *AtTIFY1/AtZIM* is the first identified *TIFY* gene in plants, and overexpression of *AtTIFY1/AtZIM* promotes the elongation of the petiole and hypocotyl, which is independent of gibberellin and brassinosteroids [4, 23]. AtZML1/AtTIFY2b and AtZML2/AtTIFY2a are important components of the CRYPTOCHROME1- (cry1-) mediated response to excess light [24]. In tomato, SlJAZ2 was described as an important regulator of the transition from vegetative growth to reproductive growth [18]. In addition, many *JAZ* genes were found to play key roles in JA signal transduction and participate in the regulation of various developmental processes and responses to biotic and abiotic stresses in plants. For example, OsTIFY3/OsJAZ1 can regulate floral development and root elongation by interacting with OsMYC2 and OsCOI1b in rice, and substitution or deletion of the core segments of OsJAZ1 can affect the specificity and sensitivity of JA signaling during flower and root development [13, 25]. In addition, OsJAZ1 was identified as a negative regulator of rice drought resistance, partially by regulating the JA and ABA signaling pathways [14]. Another rice JAZ member, OsJAZ8, can confer resistance to rice bacterial blight by regulating JA-responsive volatile compounds [26]. Additionally, rice plants expressing OsJAZ8 and OsJAZ8*Δ*C (C-terminal truncated) under the control of the salt-inducible ZOS3-11 promoter exhibited higher tolerance to salt stress in early stages [27].

Recently, genome-wide surveys of the *TIFY* gene family have been conducted in various plant species such as *Brachypodium distachyon* [28], maize [29], moso bamboo (*Phyllostachys edulis*) [30], pigeon pea (*Cajanus cajan*) [31], *Brassica rapa* [5], tomato (*Solanum lycopersicum*) [32], *Gossypium* species [33], wheat (*Triticum aestivum*) [16], poplar (*Populus trichocarpa*) [6, 34], and pear (*Pyrus pyrifolia*) [35]. However, no genome-wide information of this gene family is available in watermelon, an important agricultural crop susceptible to various biotic and abiotic stresses during growth and development [36].

In this study, we predicted and classified the *TIFY* family genes of watermelon and analyzed their distribution patterns, phylogenetic relationships, and protein and gene structures. In addition, we examined the expression profiles of several *ClTIFY* genes in various tissues during fruit development of watermelon as well as under diverse abiotic stresses. Our findings lay a solid foundation for further understanding the role of *TIFY* genes in the growth and development of watermelon.

## 2. Materials and Methods

### 2.1. Genome-Wide Identification of *TIFY* Genes in Watermelon

To identify the *TIFY* family members in watermelon, the hidden Markov model (HMM) profiles of the TIFY domain (PF06200), Jas domain (PF09425), and CCT domain (PF06203) were obtained from the Pfam database (http://pfam.sanger.ac.uk/), and these domains were used as queries to search the watermelon genome database (http://cucurbitgenomics.org/organism/1) using HMMER 3.0 software (http://hmmer.org/) with an *E* value cutoff of 1*e*^−5^. *Arabidopsis* and rice TIFY protein sequences were also used as queries to obtain the watermelon TIFYs by BlastP through searching the watermelon genome database with a cutoff *E* value of 1*e*^−5^. According to previous reports [2, 37], *Arabidopsis* and rice TIFY protein sequences were downloaded from the *Arabidopsis* genome database at TAIR (The *Arabidopsis* Information Resource, https://www.arabidopsis.org/) and from the rice genome database at RGAP (Rice Genome Annotation Project, http://rice.plantbiology.msu.edu/), respectively. The nonredundant sequences were subsequently confirmed with the Pfam database and the SMART database (http://smart.embl-heidelberg.de/).

### 2.2. Protein Properties, Sequence Analyses, and Phylogenetic Tree Construction

The biochemical features including isoelectric point (pI) and molecular weight (MW) of watermelon TIFY proteins were calculated with the ProtParam tool (http://web.expasy.org/protparam/). Conserved motifs were identified using the MEME tool (http://meme-suite.org/tools/meme) with the maximum number of motifs set as 10. The exon-intron structures of watermelon *TIFY* genes were displayed by the GSDS tool (http://gsds.cbi.pku.edu.cn) based on the alignment of coding region sequences (CDS) with the corresponding genomic sequences. For phylogenetic tree construction, TIFY protein sequences from watermelon, tomato, rice, and *Arabidopsis* were aligned by Clustal Omega with default parameters. Then, the alignments of protein sequences were used to construct the phylogenetic tree by MEGA 7.0 using the Neighbor-Joining (NJ) method, with parameters of 1,000 bootstrap replicates and pairwise deletion.

### 2.3. Chromosomal Location and Duplication Analysis

The genetic positions of watermelon *TIFY* genes on chromosomes were obtained from the watermelon genome database, and the MapChart software was used to present the chromosomal positions and relative distance of *ClTIFY* genes on the basis of their ascending order of physical position (bp). Gene duplications were conducted using multiple collinear scanning toolkit (MCScanX) software with the default parameters as previously reported [38].

### 2.4. *In Silico* Expression Analysis of *ClTIFY* Genes

The inbred line 97103 strand-specific RNA-seq of both the flesh and rind at four pivotal stages of fruit development (10 days after pollination, 10 DAP; 18 DAP; 26 DAP; and 34 DAP) was analyzed [39]. Fragments per kilobase of exon model per million mapped (FPKM) values were log_2_-transformed and heat maps with hierarchical clustering were plotted using the OmicShare tools (http://www.omicshare.com/tools).

### 2.5. Plant Materials and Treatments

Watermelon (*Citrullus lanatus* L. Xinong 8) plants were cultured in a growth room (25°C/19°C, 12 h light/12 h dark, 200 mol *μ*m^−2^ s^−1^). At the four-leaf stage, watermelon seedlings grown in hydroponics with Hoagland's solution were treated with JA and abiotic stresses. For JA treatment, the leaves were sprayed with 100 *μ*M methyl jasmonate (MeJA) solution. For abiotic stress treatments, the plants were exposed to 200 mM NaCl solution for 24 h (salt stress), or under 20% PEG-6000 (*w*/*v*) for 24 h (drought stress) under the same photoperiod and light conditions. Untreated seedlings were used as the controls. The leaves were sampled at different time points (0 h, 1 h, 3 h, 9 h, and 24 h) after treatment. For the analysis of the transcripts of *TIFY* genes in different tissues of watermelon, the roots, stems, mature leaves, stem apexes, and fruits were sampled separately from 8-week-old watermelon plants. All samples were collected and immediately frozen in liquid nitrogen and then stored in -80°C prior to RNA extraction.

### 2.6. RNA Extraction and Quantitative Real-Time PCR (qRT-PCR)

Total RNA was extracted using the total RNA Miniprep Kit (Axygen Biosciences, Union City, CA, USA) according to the manufacturer's instructions. Single-stranded cDNAs were synthesized following the manufacturer's instructions (ReverTra Ace qPCR RT Kit, Toyobo, Japan). The gene-specific primers are shown in Supplementary [Supplementary-material supplementary-material-1]. qRT-PCR was performed with the iCycler iQ™ Real-Time PCR Detection System (Bio-Rad, Hercules, CA, USA). The PCR was run at 95°C for 3 min, followed by 40 cycles of 30 s at 95°C, 30 s at 58°C, and 1 min at 72°C. The watermelon *Actin* gene (Cla007792) was used as an internal control. Each treatment was performed with three independent biological replicates and three technical replicates. Relative expression levels were calculated as described previously [40].

### 2.7. Statistical Analysis

All statistical analyses were conducted using the SPSS18 statistical package (Chicago, IL, USA). The data were subjected to one-way analysis of variance (ANOVA), and means were compared by Tukey's multiple comparisons test. *P* < 0.05 was accepted as significant, and the differences between treatment means are indicated by different letters.

## 3. Results

### 3.1. Identification and Characterization of TIFY Family Members in Watermelon

A total of 15 *ClTIFY* family genes were identified in the watermelon genome, which were further examined using SMART and PFAM to confirm that their coding protein sequences contain the TIFY, Jas, ZIM, and PPD domains. The results revealed that these 15 *ClTIFY* genes consisted of eight *ClJAZs*, four *ClZMLs*, two *ClTIFYs*, and one *ClPPD*. These genes were named according to their chromosomal positions and the domains that they contained as described in other plant species [6, 34, 35]. The amino acid sequences of TIFY family members varied from 118 aa (ClJAZ1) to 450 aa (ClTIFY2) in length, from 13.05 kDa (ClJAZ1) to 47.43 kDa (ClTIFY2) in theoretical MW, and from 4.73 (ClZML1) to 10.56 (ClJAZ5) in pI values (Table 1).

### 3.2. Phylogenetic Characterization of Watermelon *TIFY* Gene Family

**Figure 1:**
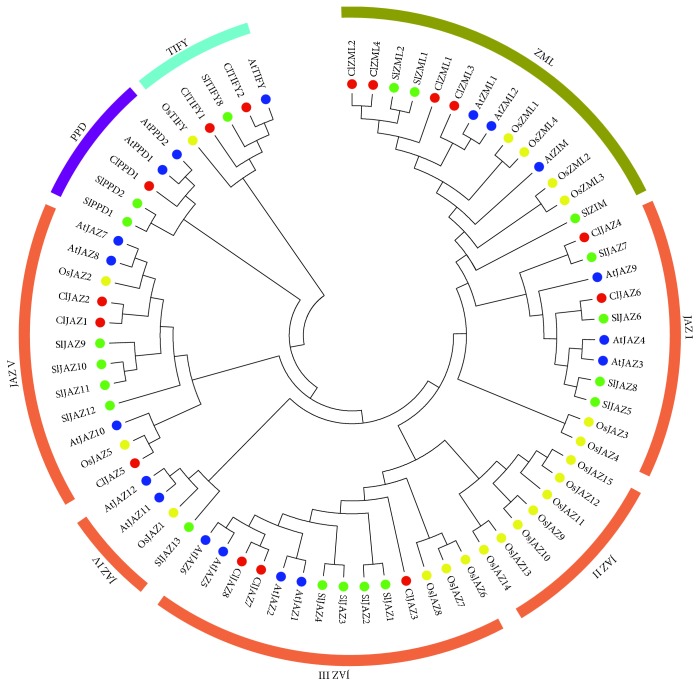
Phylogenetic relationships of TIFY family proteins of watermelon, tomato, rice, and *Arabidopsis*. The phylogenetic tree was created by MEGA 7.0 using the NJ method with 1000 bootstrap replicates.

To study the evolutionary relationship of the watermelon *TIFY* gene family, a phylogenetic tree was created from the 72 TIFY protein sequences, including 18 in *Arabidopsis*, 20 in rice, 19 in tomato, and 15 in watermelon. As a result, these TIFY proteins could be classified into four subfamilies: JAZ, ZML, TIFY, and PPD (Figure 1), among which the JAZ subfamily was the largest and could be further divided into five groups (JAZ I-V). It is noteworthy that the JAZ group I was close to the ZML subfamily, which was composed of ZIM and ZML proteins including four in watermelon, four in rice, three in *Arabidopsis*, and three in tomato, respectively. Three JAZ groups (I, III, and V) comprised JAZ proteins from *Arabidopsis*, rice, tomato, and watermelon; JAZ group IV included JAZ proteins from *Arabidopsis*, rice, and tomato; whereas JAZ group II only contained OsJAZs (Figure 1). The PPD proteins constituted a unique clade, including two members from *Arabidopsis* (AtPPD1 and AtPPD2), two from tomato (SlPPD1 and SlPPD2), and only one from watermelon (ClPPD1). The TIFY subfamily consisted of two TIFY proteins from watermelon, and one TIFY protein from each of *Arabidopsis*, rice, and tomato. It should be noted that the watermelon ClTIFY proteins were more closely related to the TIFY proteins from *Arabidopsis* and tomato than to those from rice (Figure 1).

### 3.3. Protein Architecture and Conserved Domain Analysis of TIFYs in Watermelon

The protein sequence analysis showed that all the watermelon TIFY proteins contained the TIFY domain (PF06200), with the conservation at the amino acid level of TIFYXG, except for ClZMLs, whose TIFY domain sequences were TLS(F/Y)XG (Table 1; [Supplementary-material supplementary-material-1]). In addition, thirteen of these 15 watermelon TIFY proteins (except for ClTIFY1 and ClTIFY2) possessed the Jas domain (PF09425). The conserved sequences of ClJAZs and ClZMLs were SLXRF(L/F)(E/Q)KRKXRX_5_PY and SLXRFR(E/Q)KRKXRX_7_Y, while ClPPD contained a truncated Jas motif lacking the conserved P and Y residues ([Supplementary-material supplementary-material-1]). Moreover, the GATA zinc-finger (ZnF_GATA, PF00320) domain was represented by CX_2_CX_20_CX_2_C in all the four ClZML proteins ([Supplementary-material supplementary-material-1]), which was also described in *Arabidopsis* [23] and pigeon pea [31].

**Figure 2:**
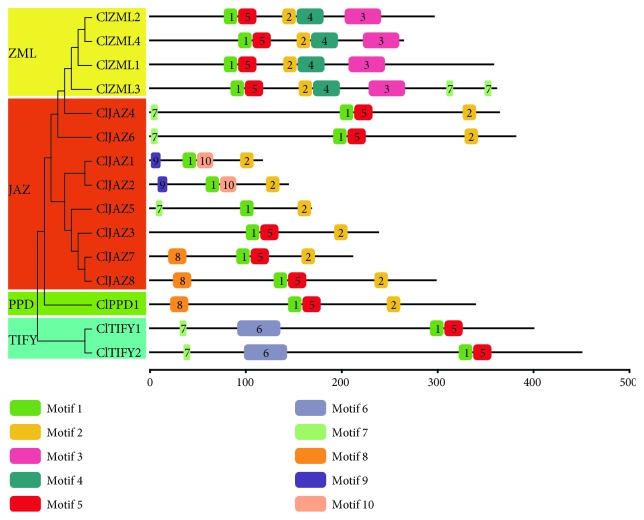
Conserved domains of ClTIFYs based on the evolutionary relationship. Different motifs are presented by different colors, and the lengths of the motifs in each protein are shown proportionally.

To further explore the diversity of the conserved domains of watermelon TIFY proteins, the MEME online tool was employed, and 10 conserved motifs were identified and named as motifs 1–10. Among them, motifs 1 and 5 or motifs 1 and 10 composed the TIFY domain, and motif 2 was annotated as the Jas domain (Figure 2). Nearly all watermelon TIFY proteins contained motifs 1 and 2, with the exception of ClTIFY1 and ClTIFY2, both of which lacked motif 2 but harbored an additional motif 6. In addition, motif 3 was annotated as the ZnF_GATA domain, motifs 2 and 4 made up the CCT domain, and both domains were only present in the ClZML proteins. In addition, both ClJAZ1 and ClJAZ2 contained motif 9 at their N-terminus, which was associated with a classical EAR motif (LXLXL) (Figure 2; [Supplementary-material supplementary-material-1]). Moreover, some watermelon TIFY proteins had unique motifs. For example, motif 8 was observed in ClJAZ7, ClJAZ8, and ClPPD1 but was absent in other watermelon TIFY proteins (Figure 2).

### 3.4. Gene Structure Analysis of *ClTIFY* Genes

**Figure 3:**
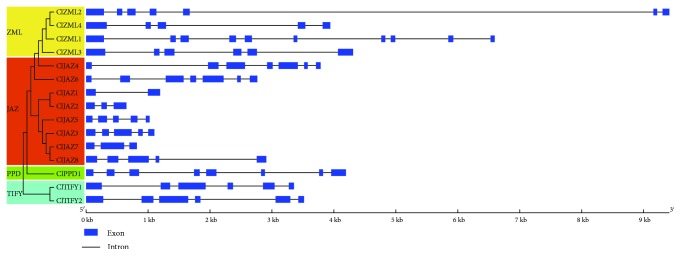
Exon-intron structures of *ClTIFY* genes based on the evolutionary relationship. Blue boxes and black lines represent the exons and introns, respectively.

To understand the structural components of the *ClTIFY* genes, the gene structures of the *ClTIFY* genes were analyzed by alignment with the CDS sequences and corresponding genomic DNA sequences. The results revealed that the *ClTIFY* genes contained 1–9 introns, and the number of introns in each subfamily varied. For example, in the JAZ subfamily, *ClJAZ4* and *ClJAZ6* had six introns; *ClJAZ3*, *ClJAZ5*, and *ClJAZ8* harbored four introns; *ClJAZ2* and *ClJAZ7* contained two introns; while *ClJAZ1* possessed only one intron (Figure 3). It can be observed that all *ZML* subfamily genes have longer genomic sequences than the *TIFY* genes of other subfamilies, and the intron number of *ClZMLs* was 4–9. However, some members in the same subfamily shared similar intron numbers but with different intron lengths, such as *ClJAZ4* and *ClJAZ6*, *ClJAZ3* and *ClJAZ5*, and *ClTIFY1* and *ClTIFY2* (Figure 3).

### 3.5. Chromosome Distribution and Duplication Analysis of *ClTIFY* Genes

**Figure 4:**
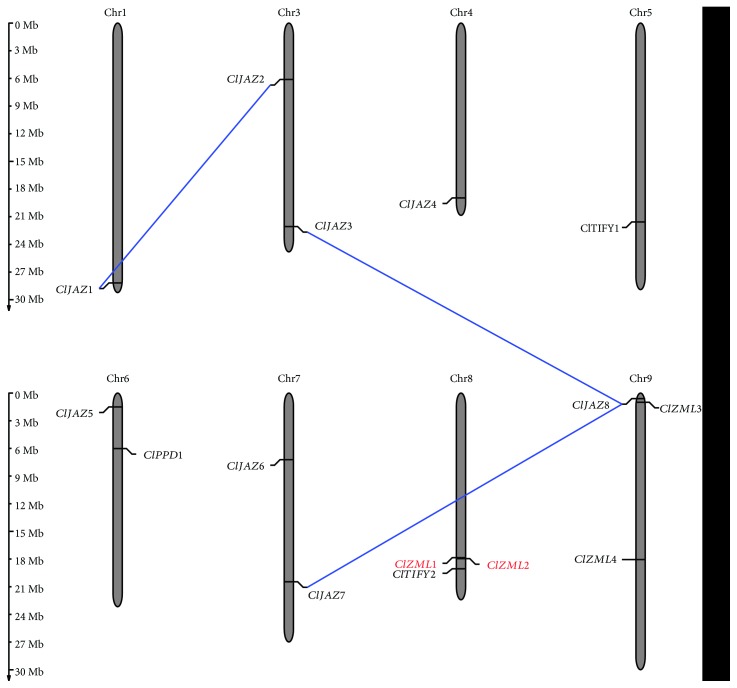
Chromosomal distribution of *ClTIFY* genes. The *ClTIFY* genes located in duplicated chromosomal segments are connected by broken lines. Only those chromosomes containing *ClTIFY* genes are represented. The tandemly duplicated genes are marked in red.

The 15 *ClTIFY* genes were distributed among eight chromosomes, with chromosomes 8 and 9 containing the most genes (three genes for each), followed by chromosomes 3, 6, and 7 (two genes for each), while chromosomes 1, 4, and 5 contained the fewest *ClTIFY* genes (one gene for each) (Figure 4). The segmental and tandem duplication events among the *ClTIFY* genes were further determined. As a result, three paralogous gene pairs (*ClJAZ1*/*ClJAZ2*, *ClJAZ3*/*ClJAZ8*, and *ClJAZ7*/*ClJAZ8*) were found to be related to segmental duplication events, while only one tandem duplication event was identified on chromosome 8 (*ClZML1*/*ClZML2*).

### 3.6. Tissue-Specific Expression Profiles of the *ClTIFY* Genes

**Figure 5:**
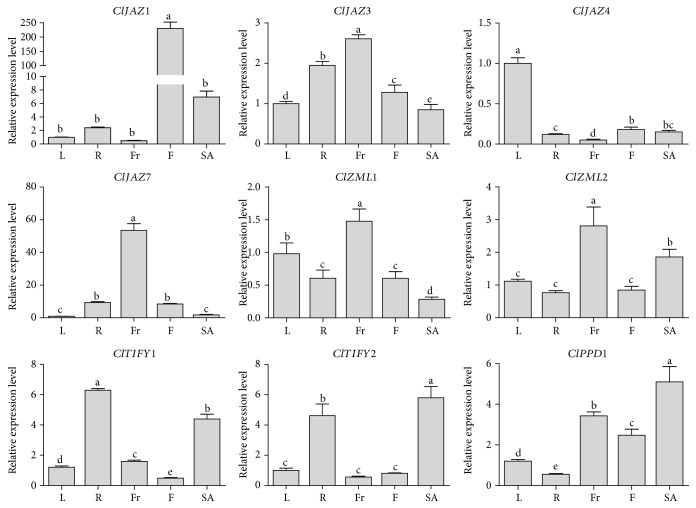
qRT-PCR analysis of the expression patterns of nine *ClTIFY* genes in different tissues of watermelon. L: leaves; R: roots; Fr: fruits; F: flowers; SA: stem apexes. The vertical axis represents relative expression levels and error bars indicate standard deviation (SD) of three independent experiments. Columns with different letters are significantly different (*P* < 0.05).

To gain insights into the expression profiles of the *ClTIFY* genes in various tissues, qRT-PCR analysis was carried out to examine the expression of nine selected *ClTIFY* genes in leaves, roots, fruits, flowers, and stem apexes. The qRT-PCR data revealed the preferential expression of these *ClTIFY* genes (Figure 5). Among them, *ClJAZ1*, *ClJAZ4*, and *ClJAZ7* were highly and preferentially expressed in flowers, leaves, and fruits, respectively. In addition, *ClJAZ3*, *ClZML1*, and *ClZML2* had higher expression in fruits than in other tissues. Interestingly, two TIFY subfamily members, *ClTIFY1* and *ClTIFY2*, were highly expressed in roots and stem apexes (Figure 5). Additionally, *ClPPD1* expression was also high in stem apexes, but relatively lower in fruits, flowers, and other tissues. The results showed that the *ClTIFY* genes had overlapping but spatially varying expression, indicating that they may play important roles in specific tissues.

### 3.7. Characterization of the Expression of *ClTIFYs* during Watermelon Fruit Development

**Figure 6:**
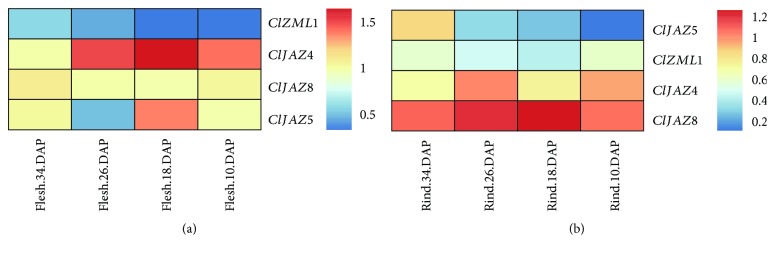
Cluster analysis of the expression of four *ClTIFY* genes during the development of flesh and rind at different stages. The expression of *TIFY* genes was determined from the RNA-seq data. Scale bars on the right of each heat map represent log_2_FPKM-transformed (treatment/control) values. Blue, yellow, and red colors indicate low, regular, and high signal intensity, respectively.

The expression profiles of *ClTIFYs* during fruit development in watermelon were analyzed according to the transcriptome data from a previous study [39]. The results showed that four *ClTIFY* genes were differentially expressed during watermelon flesh and rind development (Figure 6). During flesh development, *ClZML1* showed low expression all the time, while *ClJAZ4* showed an observable accumulation of transcripts. During the development of rind, *ClJAZ4* and *ClJAZ8* exhibited specifically higher expression at some time points, while *ClJAZ5* showed lower transcripts at all stages of rind development (Figure 6). These findings indicated that these genes might play a role in the fruit development of watermelon.

### 3.8. Expression Profiles of *ClTIFY* Genes in response to Salt, Drought, and JA Treatments

**Figure 7:**
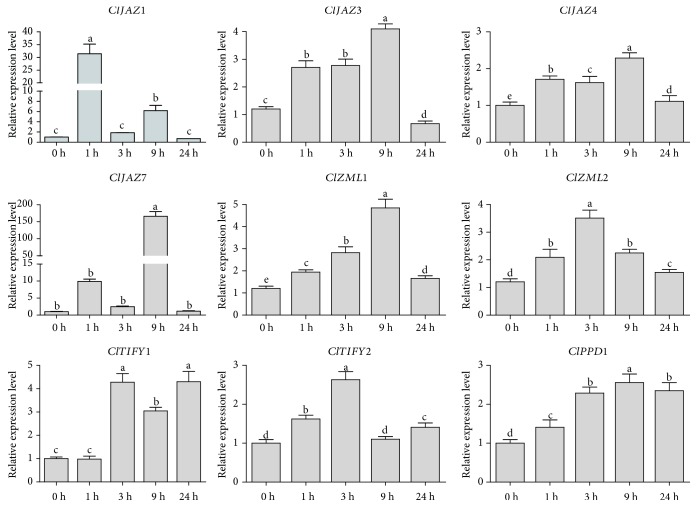
Differential expression detected for nine *ClTIFY* genes in response to drought treatment at 0 h, 1 h, 3 h, 9 h, and 24 h. The relative expression levels of the selected *ClTIFY* genes were examined by qRT-PCR. Error bars denote the SD calculated from three independent experiments. Columns with different letters are significantly different (*P* < 0.05).

To gain more insights into the roles of *ClTIFY* genes in response to hormone and various stresses, the expression of nine selected *ClTIFY* genes was analyzed by qRT-PCR under various stress treatments including salt, drought, and JA. The results suggested that most of the genes exhibited obvious variations in expression under these treatments. Under drought stress, the expression of all detected *ClTIFY* genes was significantly upregulated (Figure 7). Among them, *ClJAZ7* and *ClJAZ1* showed 166- and 31-fold increases at specific time points after drought treatment, respectively, while the other seven *ClTIFY* genes exhibited 2- to 5-fold increases in expression compared with the control (Figure 7).

**Figure 8:**
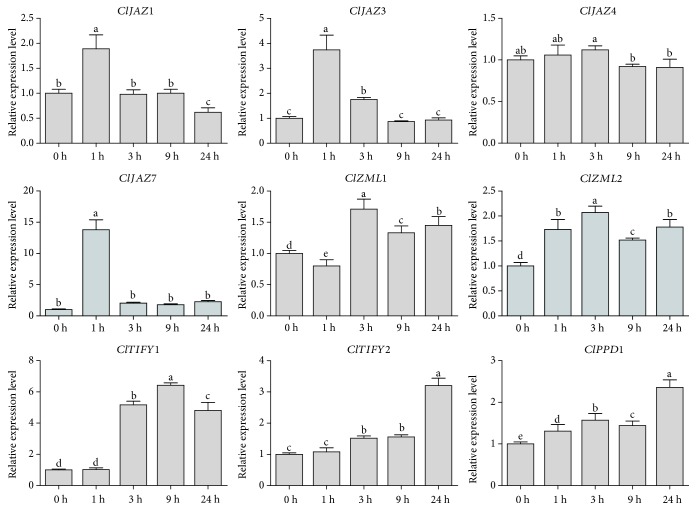
Differential expression detected for nine *ClTIFY* genes in response to salt treatment at 0 h, 1 h, 3 h, 9 h, and 24 h. Bars represent mean value ± SD of three independent experiments. Columns with different letters are significantly different (*P* < 0.05).

It was observed that nearly all the detected *ClTIFY* genes were highly induced by salt treatment, with the exception of *ClJAZ4*, which seemed to be insensitive to salt treatment (Figure 8). Notably, the transcripts of *ClJAZ1*, *ClJAZ3*, and *ClJAZ7* displayed more dramatic increases at 1 h than at other time points after treatment (Figure 8), implying that they play vital roles in the response of watermelon to salt stress in earlier periods.

**Figure 9:**
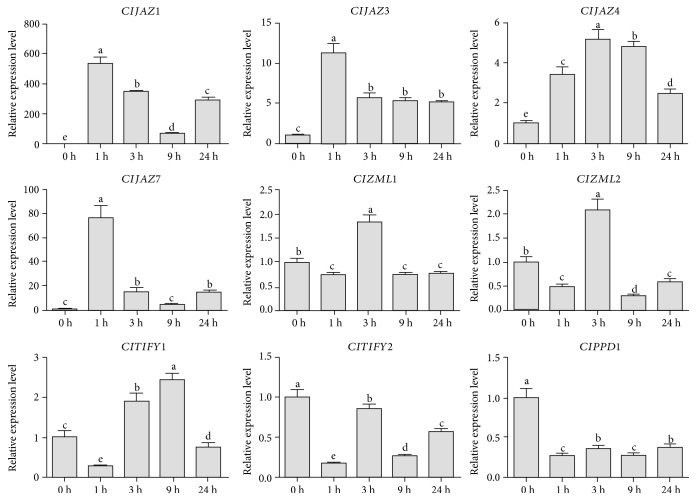
Differential expression detected for nine *ClTIFY* genes in response to JA treatment at 0 h, 1 h, 3 h, 9 h, and 24 h. Bars represent mean value ± SD of three independent experiments. Columns with different letters are significantly different (*P* < 0.05).

Upon JA treatment, eight out of the nine selected *ClTIFY* genes exhibited significantly upregulated expression, except for *ClPPD1*, whose expression was significantly reduced at all time points (Figure 9). Among the upregulated *ClTIFY* genes, *ClJAZ1* and *ClJAZ7* were the most significantly induced ones, exhibiting 533- and 77-fold changes, respectively.

## 4. Discussion

In the present study, we systematically identified 15 *TIFY* family genes from the watermelon genome, including eight *ClJAZs*, four *ClZMLs*, two *ClTIFYs*, and one *ClPPD*. Since only one *PPD* gene was present in the watermelon genome, the number of *TIFY* genes in watermelon was smaller than that in other plant species, whose numbers of *TIFY* genes range from 18 to 54, such as *Arabidopsis* (18) [37], rice (20) [2], *Brachypodium distachyon* (21) [28], pear (21) [35], poplar (24) [6, 34], maize (30) [29], *Brassica rapa* (36) [5], wheat (49) [16], *Gossypium hirsutum* (50), and *G. barbadense* (54) [33]. This difference might be attributed to gene duplication events, including large segmental duplications and small-scale tandem duplications, which have been proven to play a key role in the expansion and function diversification of genes in *TIFY* gene family [2, 28, 41]. In this study, only three segmental duplications and one tandem duplication were observed (Figure 4), and the number is much smaller than that in other plants, indicating that the identified *TIFY* genes are indispensable for the growth and development of watermelon.

Phylogenetic analysis showed that watermelon TIFY proteins can be divided into four subfamilies (JAZ, ZML, TIFY, and PPD), and the JAZ subfamily can be further classified into five groups (Figure 1). It was observed that some JAZ proteins were clustered in monocot- or eudicot-specific patterns, suggesting that these JAZ proteins might have predated the divergence between monocotyledonous and dicotyledonous plants (Figure 1). Multiple sequence alignments showed that the Jas motif of ClJAZs is strikingly similar to the special consensus sequence of SLX_2_FX_2_KRX_2_RX_5_PY in other plants [3, 5], which has been reported to participate in the formation of the JA-Ile-COI1-JAZ complex [42]. However, the sequence of conserved Jas domains from ClZML proteins was SLXRFR(E/Q)KRKXRX_7_Y, which is slightly different from the characteristic sequence ([Supplementary-material supplementary-material-1]), suggesting that ClJAZs and ClZMLs might play different roles in regulating jasmonate responses in watermelon. We further detected 10 conserved motifs by MEME, and some conserved motifs were unique to specific subfamilies in watermelon TIFY proteins (Figure 2), which may contribute to the function diversification of TIFY proteins. Considering that the exon/intron organization can provide additional clues for understanding the evolutionary relationships among gene families [43], the gene structures of *ClTIFY* genes were determined in this study. Similar to the case in other plants [1, 31], *ClZML* genes have longer structures and a larger number of introns than other genes (Figure 3). Besides, *ClZML* genes exhibited a relatively similar pattern of exon/intron organization but had different intron numbers, indicating that gain or loss of introns occurred during the evolution of *ClZML* genes, which may lead to the functional divergence [43, 44]. Moreover, the JAZ subfamily genes exhibited the highest variability in exon/intron organization, while *ClJAZ4* and *ClJAZ6* had the same number of introns and similar lengths of exons (Figure 3), suggesting that these two genes are highly conserved during evolution and may have similar functions.

The signal molecule JA plays vital roles in plant growth, development, and responses to environmental stresses. Previous studies have revealed that the *TIFY* genes play vital roles in various biological processes of plants, such as petiole and hypocotyl elongation [4], lamina size and curvature [9, 11], flower development [13, 25, 45], and seed germination [46]. *TIFYs* may regulate plant development through the JA signaling pathway. For example, some JAZ proteins can interact with the WD-Repeat/bHLH/MYB complexes to repress JA-mediated trichome initiation and anthocyanin accumulation in *Arabidopsis* [47]. In *Arabidopsis*, JAZ4 and JAZ8 competitively interact with WRKY57 to mediate JA-induced leaf senescence [48]. In this study, all the selected *ClTIFY* genes were regulated by JA and exhibited preferential expression in specific tissues (Figures 5 and 9), suggesting that these genes play specific roles in regulating the normal development of plants probably through the JA signaling pathway. It is noteworthy that a majority of *ClTIFY* genes were highly expressed in flowers and fruits (Figure 5), and four *ClTIFY* genes might play crucial roles in watermelon fruit development (Figure 6), suggesting that *ClTIFY* genes may function in the development of the flower and fruit of watermelon. It should be noted that abiotic stress can increase the JA content, and *TIFY* genes were also shown to play vital roles in response to abiotic stresses through the JA signaling pathway. For example, overexpression of apple *MdJAZ2* in *Arabidopsis* decreased JA sensitivity and increased the tolerance to salt and drought stresses during seedling development [49]. Moss PnJAZ1 acts as a repressor to mediate the crosstalk between JA and ABA signaling pathways, and thus increases tolerance to salt stress [21]. In this study, the vast majority of the detected *ClTIFYs* exhibited differential accumulations under salt and drought stresses. Similar results were also reported in other plants, such as apple [41] and *B. rapa* [5]. In addition, nearly all the detected *ClTIFYs* were upregulated by JA treatment, with the exception of *ClPPD1*, whose expression was downregulated (Figure 9). It is worth noting that *ClJAZ1* and *ClJAZ7* showed the most remarkable increases in expression under JA and drought treatments (Figures 7 and 9), indicating that they may play essential roles in regulating the response to drought stress by the JA-mediated signaling pathway. ClJAZ1 was clustered together with AtJAZ7 in the JAZ V group (Figure 1), and overexpression of *AtJAZ7* was found to confer drought tolerance in *Arabidopsis* [50]. Under salt treatment, *ClJAZ7* exhibited the highest expression among the detected *ClTIFYs* (Figure 8), demonstrating that it may play a major role in salt stress response. Similarly, overexpression of *OsTIFY11a/OsJAZ9* in rice resulted in significantly enhanced tolerance to salt and dehydration stresses, and suppression of *OsJAZ9* resulted in reduced salt tolerance through the regulation of JA signaling [2, 15]. Further studies of these *ClTIFY* genes are needed to unravel their regulatory roles in the development of watermelon and in abiotic stress response through the JA signaling pathway.

## 5. Conclusions

In this study, a total of 15 *TIFY* genes were identified in the watermelon genome, including eight *ClJAZs*, four *ClZMLs*, two *ClTIFYs*, and one *ClPPD*. The analysis of qRT-PCR and RNA-seq data revealed that some *TIFY* genes play organ-specific roles. Expression patterns of the nine selected *TIFY* genes in response to JA and abiotic stress indicated that they may be involved in abiotic stress response by the JA signaling pathway. Our findings lay a foundation for a further functional characterization of the *TIFY* family genes in watermelon and a clarification of how *TIFY* genes can be utilized for the improvement of watermelon via biotechnological strategies.

## Figures and Tables

**Table 1:** *ClTIFY* family genes identified in watermelon.

Gene name	Gene ID	Map position (bp)	CDS length	Protein length (aa)	MW (kDa)	pI	TIFY motif
*ClJAZ1*	Cla009781	Chr1:32842754:32843948	357	118	13.05	10.13	TIFYNG
*ClJAZ2*	Cla019575	Chr3:7148182:7148833	438	145	16.37	7.64	TIFYNG
*ClJAZ3*	Cla011143	Chr3:25698497:25699599	795	239	25.35	10.29	TIFYAG
*ClJAZ4*	Cla018445	Chr4:22124533:22128318	720	365	38.73	9.58	TIFYAG
*ClTIFY1*	Cla020951	Chr5:25205424:25208778	1203	400	42.65	9.18	TIFYGG
*ClJAZ5*	Cla001487	Chr6:1759629:1760654	510	169	18.51	10.56	TIFYNG
*ClPPD1*	Cla007531	Chr6:7067544:7071737	1023	340	37.36	7.84	TIFYCG
*ClJAZ6*	Cla003391	Chr7:8397563:8400326	1149	382	39.65	9.05	TIFYGG
*ClJAZ7*	Cla012536	Chr7:23895841:23896660	639	212	23.22	9.43	TIFYDG
*ClZML1*	Cla022146	Chr8:20879791:20886385	1053	350	38.90	4.73	TLSFEG
*ClZML2*	Cla022147	Chr8:20889642:20899056	894	297	31.98	6.76	TLSFRG
*ClTIFY2*	Cla022284	Chr8:22248851:22252368	1353	450	47.43	8.91	TIFYGG
*ClJAZ8*	Cla015533	Chr9:695779:698687	900	299	32.22	9.80	TIFYAG
*ClZML3*	Cla015488	Chr9:1150618:1154927	1089	362	40.78	8.18	TLSYQG
*ClZML4*	Cla012926	Chr9:21054263:21058204	798	265	28.47	5.45	TLSFRG

## Data Availability

The original data of the TIFY family genes are available from the watermelon genome database (http://cucurbitgenomics.org/organism/1).

## Supplementary Materials

Supplementary 1 Supplementary Fig. S1 Alignment of the amino acid sequences of watermelon TIFY proteins.

Supplementary 2 Supplementary Table S1 Primer sequences used in qRT-PCR.

Supplementary 3 Supplementary Table S2 Sequences and lengths of ClTIFY motifs.
