# Supplementary figures and images for: Comprehensive Analysis of TIFY Transcription Factors and Their Expression Profiles under Jasmonic Acid and Abiotic Stresses in Watermelon
Source: Int J Genomics. 2019 Oct 1;2019:6813086. doi: 10.1155/2019/6813086 (PMC6791283; doi:10.1155/2019/6813086)

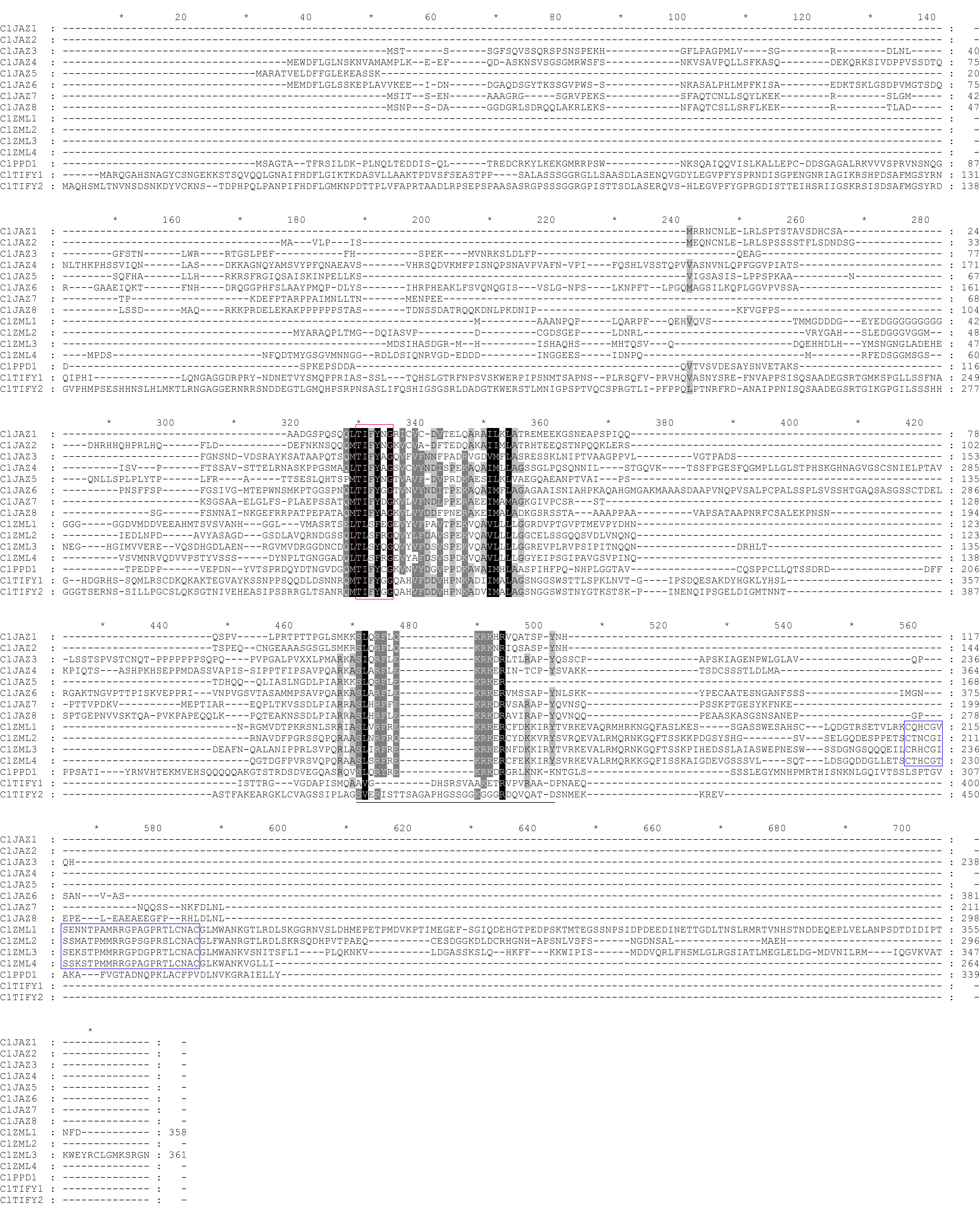

Supplement: Supplementary 1 — Supplementary Fig. S1 Alignment of the amino acid sequences of watermelon TIFY proteins. [file 6813086.f1.tif]
