# Supplementary material for: Comprehensive Analysis of TIFY Transcription Factors and Their Expression Profiles under Jasmonic Acid and Abiotic Stresses in Watermelon
Source: Int J Genomics. 2019 Oct 1;2019:6813086. doi: 10.1155/2019/6813086 (PMC6791283; doi:10.1155/2019/6813086)
Supplement: Supplementary 3 — Supplementary Table S2 Sequences and lengths of ClTIFY motifs. [file 6813086.f3.doc]

**Table S2.** Sequences and lengths of ClTIFY motifs.

| **Motif** | **Conserved amino acid sequences** | **Site** | **Width** |
| --- | --- | --- | --- |
| 1 | QMTIFYAGQVYVFDD | 15 | 15 |
| 2 | RKASLQRFLEKRKER | 13 | 15 |
| 3 | ETSCTHCGISEKSTPMMRRGPAGPRTLCNACGLMWANKG | 4 | 39 |
| 4 | CFDKKIRYSVRKEVALRMQRKKGQFTSSK | 4 | 29 |
| 5 | VPPEKAQAIMLLAGGGEVPS | 12 | 20 |
| 6 | HLEGVPFYGPRGDISGPENGNRIIGIKRSIPDSAFMGSYRDGIPHI | 2 | 46 |
| 7 | IEHDFLGL | 6 | 8 |
| 8 | EKSNFAQTCNLLSRYLKEKR | 3 | 20 |
| 9 | MERNCNLELRL | 2 | 11 |
| 10 | TEDQAKAIJKLATREMEE | 2 | 18 |
